# Assessing health-related quality of life among cancer survivors during systemic and radiation therapy in Bangladesh: a cancer-specific exploration

**DOI:** 10.1186/s12885-023-11670-z

**Published:** 2023-12-07

**Authors:** Md. Shahjalal, Marufa Sultana, Jeff Gow, Mohammad Enamul Hoque, Sabuj Kanti Mistry, Ahmed Hossain, Rashidul Alam Mahumud

**Affiliations:** 1 Department of Public Health, North South University, Dhaka, Bangladesh; 2 Research Rats, Dhaka, Bangladesh; 3 Deakin Health Economics, Institute for Health Transformation, School of Health and Social Development, Faculty of Health, Deakin University, Geelong, VIC 3220 Australia; 4 School of Business and Centre for Health Research, University of Southern Queensland, Toowoomba, QLD Australia; 5 School of Accounting, Economics and Finance, College of Law and Management Studies, University of KwaZulu-Natal, Durban, South Africa; 6 Faculty of Medicine and Health, Health Economics and Health Technology Assessment Unit, NHMRC Clinical Trials Centre, The University of Sydney, Camperdown, New South Wales (NSW) Australia; 7 School of Population Health, University of New South Wales, Sydney, Australia; 8 College of Health Sciences, University of Sharjah, Sharjah, UAE

**Keywords:** Cancer, HRQoL, Cancer survivor, Systemic therapies, Radiotherapy, Bangladesh

## Abstract

**Background:**

Evaluating the effects of cancer diagnosis and treatment on a patient’s overall well-being is crucial and health-related quality of life (HRQoL) is a reliable metric for assessing this impact. Little is known about HRQoL among cancer survivors across various stages and treatments. The study examined individual and clinical factors influencing HRQoL among cancer survivors.

**Methods:**

A cross-sectional study was conducted in two specialised cancer care hospitals in Dhaka, Bangladesh. Cancer-diagnosed adults receiving treatment at selected hospitals from January to May 2022 were enrolled. The 5-level EuroQol-5 Dimensions version (EQ-5D-5L) instrument was used to collect HRQoL data. HRQoL scores were derived using UK value sets. The investigation used a multivariable Tobit regression model to determine the association between independent variables and HRQoL scores.

**Results:**

A total of 607 adult patients were enrolled, with 55% being females and 66% aged 36 to 64 years. Reported health problems in five EQ-5D domains include mobility (11%), self-care (11%), usual daily activities (19%), pain/discomfort (21%), and anxiety/depression (46%). Patients with throat, brain, lung, blood, and liver cancer had lower utility scores. Advanced-stage cancer survivors had lower utility scores (β = -49 units, 95% codfidence interval [CI]: -0.75 to -0.22) compared to early-stage survivors. Physically inactive survivors had lower utility scores by 0.41 units (95% CI: -0.51 to -0.30) compared to their counterparts. Private hospital patients had higher utility scores, whereas patients belonged to poor socioeconomic groups scored worse than wealthier ones.

**Conclusions:**

This study highlights the impact of clinical and individual characteristics on HRQoL among cancer survivors. These findings advocate for an enhanced Bangladeshi cancer patient care model through timely interventions or programs, early detection or diagnosis, tailored treatments, and the promotion of physical activity to bolster HRQoL outcomes.

**Supplementary Information:**

The online version contains supplementary material available at 10.1186/s12885-023-11670-z.

## Introduction

Cancer poses a significant challenge worldwide and is a major barrier to increasing life expectancy [1]. According to the 2020 Global Cancer Observatory report, approximately 19.3 million new cases were reported, resulting in around 10 million cancer-related deaths [2]. The increasing burden of cancer incidence and mortality, particularly in low- and middle-income countries (LMICs), highlights the urgency of addressing rising concerns [3]. Like other LMICs, Bangladesh has seen a rise in the number of cancer cases, with 156,775 new cancer cases and 108,990 cancer-related mortality reported in 2020 [4].

High-income countries have seen higher survival rates due to high-quality and cost-effective cancer screening, timely diagnoses, improved therapeutic treatment modalities, and accessible routine follow-up care services [5]. However, many LMICs, including Bangladesh, struggle with lower cancer survival rates due to deficiencies in screening programs and suboptimal cancer management initiatives [6]. Since cancer is a chronic disease that requires persistent treatments, it can adversely impact the quality of life of cancer patients [7, 8]. The treatment phases involve a number of physical and psychosocial challenges for patients, which can affect their quality of life [9–11]. Additionally, pre-existing medical conditions/diseases coupled with adverse effects of cancer treatments can severely reduce survivors' functional capacity and overall quality of life [9, 12, 13].

Patient-centric HRQoL has emerged as an important metric and gained recognition as a measurable health outcome in clinical trials and strategies for clinical practice improvement [14–16]. Understanding patients’ HRQoL and functional capacity is vital for clinicians because this insight provides prognostic cues and sheds light on patients’ resilience to cancer treatment modalities [14]. Additionally, it helps in identifying specific challenges that might influence treatment decisions, facilitating adapted care for cancer survivors.

The EuroQol-5 dimensions instrument (EQ-5D-5L) has gained popularity as a method to assess HRQoL among cancer survivors in clinical settings [17–20]. Identifying HRQoL challenges in EQ-5D-5L domains not routinely assessed holds the potential for refining risk mitigation strategies, improving treatment procedures, and increasing survival rates [20, 21]. Therefore, HRQoL insights regarding cancer survivors are essential within clinical practices, offering a better understanding of how HRQoL is shaped by cancer site and stage during treatment. However, the specific relationship between these variables remains largely unexplored in Bangladesh. Therefore, attention to the HRQoL of cancer patients across all stages, focusing not solely on survival but also on systemic treatment-related experiences, is important.

Exploring HRQoL among cancer survivors throughout treatment holds profound implications, aligning with the Sustainable Development Goals (SDGs), particularly target 3.4 aimed at reducing premature death [22]. This exploration informs the development of clinical strategies to alleviate cancer burden, optimise functional capacity, and improve HRQoL. Furthermore, understanding how HRQoL outcomes vary with sociodemographic and clinical factors can offer insights to shape clinical practice, health policy, and appropriate healthcare interventions.

To address these imperatives, this study formulates two specific research questions (RQs):

- RQ1: What is the distribution of HRQoL among Bangladeshi cancer survivors during systemic and radiotherapy periods, categorised by cancer stages and tumor sites?
- RQ2: What person and clinical characteristics are associated with increased or decreased HRQoL?

## Methods

### Study design and settings

A cross-sectional study design was adopted to investigate HRQoL among cancer patients during systemic and radiation therapy. The survey was conducted at the outpatient departments of the two largest cancer management hospitals in Dhaka, Bangladesh. To ensure geographical diversity and maximum coverage of patient treatments within a short study period, we selected the National Institute of Cancer Research & Hospital (NICRH), a government-funded national cancer research and treatment health facility. This 300-bed tertiary care public hospital offers multidisciplinary cancer care. According to hospital records, 83,795 new patients received systemic and radiotherapy treatments from January 2018 to December 2020, coming from eight different administrative divisions in Bangladesh. To capture diverse healthcare settings and multidisciplinary cancer care, we also chose Ahsania Mission Cancer Hospital, a privately funded 50-bed tertiary care hospital that provides advanced cancer care services, and approximately 100 cancer patients visit the hospital's outpatient department each day.

The study hospitals are located in the country’s capital city, where people commonly come for treatment due to availability and potential access to better healthcare facilities for cancer.

### Study participants and survey procedures

The study population was cancer patients in Bangladesh with the following inclusion criteria: (i) age at diagnosis equal to or more than 18 years, (ii) receiving treatment, (iii) able to respond to the questions, and (iv) willing to participate in the study. Written consent was taken from each participant prior to the survey. For the illiterate or those with no education, interviewers read the consent document, explained the research details to the participants and approached adult caregivers as witnesses to attest to the prospective subject's apparent understanding and willingness to participate. Then, the subject used a tick mark instead of a signature, and a witness signed the consent form, attesting that the subject agreed to participate.

Data were collected through face-to-face interviews by trained interviewers between 1^st^ January and 30^th^ May 2022. A team of two doctors and three medical students conducted the interviews. Before data collection, 3-day training was provided to interviewers about the content of the questionnaire, ethical issues, privacy concerns, and risk management for cancer patients. Following that, the questionnaire was piloted with 24 patients and modified and finalised upon feedback. Eligible patients who were attending the hospital were approached to participate, and daily recruitment was conducted. Upon receiving consent, interviewers confirmed the patients' cancer status by reviewing diagnostic reports (biopsy, CT scan, MRI, X-ray, prescription, etc.). The required minimum sample size was 376 at 90% power, 95% confidence interval [CI] of 0.05 to 1.96, with 53.76% of patients had poor quality of life [23], and the margin of error was 5%. We had an opportunity to collect data from 607 cancer survivors through face-to-face interviews (response rate was 86%). An interview was conducted in a separate place from the patient waiting areas  or rooms. Every interview was completed within ~ 10 to15 minutes. Each completed questionnaire was spot-checked by the first author (MS) to assess the accuracy, completeness, and consistency of the data collected. The respondents were not given any financial benefits for participating in the study. Data selection, identification and inclusion flow diagram process are presented in Appendix 1.

### Outcome measures

This study examined HRQoL as the primary outcome measure using the EQ-5D-5L in English [24]. The EQ-5D-5L has been a widely used instrument for assessing HRQoL among cancer patients with poor health [20, 25, 26]. The EQ-5D-5L has superior measurement properties compared to EQ-5D-3L [21]. It contains five domains (mobility, self-care, usual activities, pain or discomfort and anxiety or depression), with each having five levels (no problems, some problems, moderate problems, severe problems or extreme problems) [27]. The instrument also rates overall health using EQ-VAS and has been translated into 130 languages [24, 27]. This instrument can be self-administered, and respondents also rate their overall health on the day of the interview on a 0–100 hash-marked, vertical visual analogue scale known as the EuroQol-visual analogue scale (EQ-VAS).

### Cancer sites and stages

Various pertinent clinical attributes were taken into account for examination, encompassing the specific site and stage of the diagnosed cancer. Detailed information regarding the diagnosed cancer's typology and its corresponding stage was garnered from the patient's comprehensive pathological diagnostic reports or treatment protocols. Among the spectrum of diagnosed cancers, prevalent categories included oral, breast, blood, pancreatic, liver, lung, brain, throat, and cervical, as well as other diverse tumor sites (such as ocular, cutaneous, osseous, penile, and ovarian malignancies).

### Covariates

The analytical explorations encompassed a range of covariates, including individual-level socioeconomic, demographic, lifestyle, and clinical variables. Sociodemographic variables include gender (male vs. female), age categories (18–35, 36–45, 46–64, and > 64 years), body-mass-index [BMI] (underweight: < 18.49 kg/m^2^, healthy weight: 18.50–24.99 kg/m^2^, overweight: 25–29.99 kg/m^2^, and obese: ≥ 30 kg/m^2^), marital status (single/ never married, married, and widowed or divorced or seperated), education (no education, primary: 1–5 years, secondary: 6–10 years, higher secondary: 10–12 years and tertiary: graduate school & above), residence (urban vs. rural), household size (< 4, 4–5 and ≥ 6 members), occupation (unemployed, employed, business, informal workers, housewife, student and other occupations), and monthly household income distributed from the lowest quintile (20% lowest quintile [Q1: poorest] to 20% highest quintile [Q5: richest]). Lifestyle aspects were accounted for through variables such as smoking status (yes vs. no), use of smokeless tobacco: betel leaf with areca nut, zarda, sadapata, and gul (yes vs. no), and adherence to physical activity recommendations: walk > 150 min per week (yes vs. no). Lastly, clinical attributes involving the cancer site and stage, along with the healthcare facility responsible for ongoing treatment (public facility vs. private facility), were encompassed.

### Statistical analysis

Descriptive statistics were presented employing frequency and percentages, or mean and standard deviation, as appropriate. Health utility scores were generated from the UK tariff corresponding to each dimension and level of the EQ-5D-5L tool [28], constituting the primary outcome variable. These mean scores were stratified across patients' distinctive attributes and cancer stages. We applied a single imputation method to replace missing data with a single predicted value for estimating health utilities, such as the mean for a given case [29]. To appraise the bivariate relationship between EQ-5D health states and cancer stage, chi-squared tests were employed, with Fisher's exact chi-squared test applied if feasible.

HRQoL data often have a limited range, constrained between a lower boundary (worst health state) and an upper boundary (best health state). Tobit models can accommodate these boundary restrictions, ensuring that the estimated coefficients and predictions stay within the feasible range of HRQoL scores. Unadjusted and adjusted multivariate tobit regression models were used to find the factors influencing patient health utility scores. In the adjusted model, predictor variables were integrated solely if any label of the predictor demonstrated significance at a risk threshold of ≤ 5% in the unadjusted regression model. This adjustment was undertaken to counteract the influence of other potential factors. The analysis was executed using the STATA-15 statistical software (Stata Corp LP, College Station, TX, USA).

## Results

### Participant’s sociodemographic characteristics

A total of 607 cancer survivors were enrolled. Among them, 55% were female, 44% aged between 46 and 64 years, 88% resided in rural areas, and 88% receiving care at a public health facility. Nearly half (47%) of cancer survivors reported no formal education and 34% belonged to economically disadvantaged households. Active workforce participation was found to be low, with 23% being unemployed, and 41% identifying as housewives. About 70% of cancer survivors engaged in regular physical activity. Common cancer diagnoses included cervical (20%), breast (18%), oral (11%), and lung (11%) cancers and 42% of patients were diagnosed with stage I (Table 1).

**Table 1:** Participant’s demographic and clinical characteristics (n = 607 patients)

Patients’ characteristics	n (%)	95% CI
**Age, years**		
18 to 35	84 (13.84)	(11.31 to 16.83)
36 to 45	130 (21.42)	(18.33 to 24.87)
46 to 64	268 (44.15)	(40.24 to 48.14)
> 64	125 (20.59)	(17.56 to 24.00)
**Gender**		
Male	275 (45.30)	(41.37 to 49.29)
Female	332 (54.70)	(50.71 to 58.63)
**Marital status**		
Never married	24 (3.95)	(2.66 to 5.84)
Married	537 (88.47)	(85.67 to 90.78)
Widowed/divorced/separated	46 (7.58)	(5.72 to 9.98)
**Education**		
No education	286 (47.19)	(43.24 to 51.19)
Primary	153 (25.25)	(21.94 to 28.87)
Secondary	94 (15.51)	(12.84 to 18.62)
Higher secondary	45 (7.43)	(5.59 to 9.81)
Tertiary	28 (4.62)	(3.21 to 6.62)
**BMI group**		
Underweight: < 18.49	100 (16.47)	(13.73 to 19.65)
Healthy weight: 18.50 to 24.99	373 (61.45)	(57.5 to 65.25)
Overweight: 25.00 to 29.99	90 (14.83)	(12.21 to 17.89)
Obese: ≥ 30	44 (7.25)	(5.43 to 9.61)
**Residence**		
Rural	534 (87.97)	(85.13 to 90.33)
Urban	73 (12.03)	(9.67 to 14.87)
**Family members**		
< 4	76 (12.52)	(10.11 to 15.41)
4 to 5	298 (49.09)	(45.12 to 53.08)
≥ 6	233 (38.39)	(34.59 to 42.33)
**Occupation**		
Unemployed	140 (23.06)	(19.88 to 26.59)
Employed	39 (6.43)	(4.73 to 8.68)
Businessperson	41 (6.75)	(5.01 to 9.05)
Housewife	248 (40.86)	(37 to 44.83)
Informal workers	40 (6.59)	(4.87 to 8.87)
Students	13 (2.14)	(1.25 to 3.66)
Other occupations	86 (14.17)	(11.61 to 17.18)
**Income quintile**		
Q1 (20% lowest)	208 (34.27)	(30.59 to 38.14)
Q2	53 (8.73)	(6.73 to 11.26)
Q3	111 (18.29)	(15.40 to 21.57)
Q4	130 (21.42)	(18.33 to 24.87)
Q5 (20% highest)	105 (17.30)	(14.49 to 20.52)
**Received COVID-19 vaccine**		
No	172 (28.34)	(24.89 to 32.06)
Yes	435 (71.66)	(67.94 to 75.11)
**Smoking history**		
No	446 (73.48)	(69.81 to 76.84)
Yes	161 (26.52)	(23.16 to 30.19)
**Smokeless tobacco users**		
No	321 (52.88)	(48.89 to 56.84)
Yes	286 (47.12)	(43.16 to 51.11)
**Physical activity** (walk > 150 min per week)		
No	184 (30.31)	(26.78 to 34.10)
Yes	423 (69.69)	(65.9 to 73.22)
**Tumor site**		
Oral	67 (11.04)	(8.78 to 13.79)
Breast	107 (17.63)	(14.79 to 20.87)
Lung	66 (10.87)	(8.63 to 13.61)
Blood	12 (1.98)	(1.12 to 3.45)
Pancreas	13 (2.14)	(1.25 to 3.66)
Liver	21 (3.46)	(2.26 to 5.25)
Cervix	121 (19.93)	(16.94 to 23.31)
Brain	12 (1.98)	(1.12 to 3.45)
Throat	16 (2.64)	(1.62 to 4.26)
Other sites	172 (28.34)	(24.89 to 32.06)
**Tumor stage**		
Stage-0	20 (3.29)	(2.13 to 5.06)
Stage-I	254 (41.85)	(37.97 to 45.82)
Stage-II	199 (32.78)	(29.16 to 36.63)
Stage-III	102 (16.80)	(14.03 to 20.00)
Stage-IV	32 (5.27)	(3.75 to 7.36)
**Ongoing treatment facility**		
Public facility	533 (87.81)	(84.95 to 90.19)
Private facility	74 (12.19)	(9.81 to 15.05)

***Abbreviation*:** *CI* Confidence interval, *BMI* Body mass index

### Health state using EQ-5D-5L dimensions by stages of cancer

Table 2 represents the various health states within the EQ-5D-5L domains, considering different cancer stages. Patients' health states deteriorated as cancer progressed, leading to significant differences in HRQoL between those with advanced-stage (stage IV) cancer and those with early-stage (stage I) cancer. For instance, patients with advanced-stage cancer reported more severe or extreme problems in all five dimensions compared to early-stage counterparts (mobility [25.00% vs. 0.00%]; self-care [28.13% vs. 10.00%]; daily activities [34.38% vs. 20.00%], pain or discomfort [34.38% vs. 10.00%], and anxiety/depression [56.26% vs. 25.00%]).

**Table 2:** Health-related quality of life measured by EQ-5D-5L utility scores by tumor stages

EQ-5D Domain	Health State	Stage-0	Stage-I	Stage-II	Stage-III	Stage-IV	Overall	*p*-value
**Mobility**	No problems	13 (65.00)	137 (53.94)	101 (50.75)	61 (59.80)	18 (56.25)	330 (54.37)	**0.010**
	Slight problems	3 (15.00)	65 (25.59)	56 (28.14)	17 (16.67)	na	141 (23.23)	
	Moderate problems	4 (20.00)	22 (8.66)	24 (12.06)	16 (15.69)	6 (18.75)	72 (11.86)	
	Severe problems	na	17 (6.69)	14 (7.04)	5 (4.90)	5 (15.63)	41 (6.75)	
	Unable/extreme problems	na	13 (5.12)	4 (2.01)	3 (2.94)	3 (9.38)	23 (3.79)	
**Self-care**	No problems	13 (65.00)	126 (49.61)	85 (42.71)	57 (55.88)	15 (46.88)	296 (48.76)	
	Slight problems	5 (25.00)	72 (28.35)	57 (28.64)	22 (21.57)	1 (3.13)	157 (25.86)	**0.007**
	Moderate problems	na	36 (14.17)	37 (18.59)	12 (11.76)	7 (21.88)	92 (15.16)	
	Severe problems	2 (10.00)	12 (4.72)	15 (7.54)	7 (6.86)	5 (15.63)	41 (6.75)	
	Unable/extreme problems	na	8 (3.15)	5 (2.51)	4 (3.92)	4 (12.50)	21 (3.46)	
**Usual activities**	No problems	11 (55.00)	96 (37.80)	62 (31.16)	45 (44.12)	14 (43.75)	228 (37.56)	
	Slight problems	3 (15.00)	73 (28.74)	59 (29.65)	23 (22.55)	2 (6.25)	160 (26.36)	**0.002**
	Moderate problems	2 (10.00)	43 (16.93)	39 (19.60)	18 (17.65)	5 (15.63)	107 (17.63)	
	Severe problems	4 (20.00)	31 (12.20)	23 (11.56)	7 (6.86)	3 (9.38)	68 (11.20)	
	Unable/extreme problems	na	11 (4.33)	16 (8.04)	9 (8.82)	8 (25.00)	44 (7.25)	
**Pain/discomfort**	No problems	4 (20.00)	39 (15.35)	44 (22.11)	22 (21.57)	4 (12.50)	113 (18.62)	**0.021**
	Slight problems	5 (25.00)	74 (29.13)	73 (36.68)	38 (37.25)	8 (25.00)	198 (32.62)	
	Moderate problems	9 (45.00)	76 (29.92)	44 (22.11)	33 (32.35)	9 (28.13)	171 (28.17)	
	Severe problems	2 (10.00)	50 (19.69)	26 (13.07)	7 (6.86)	7 (21.88)	92 (15.16)	
	Unable/extreme problems	na	15 (5.91)	12 (6.03)	2 (1.96)	4 (12.50)	33 (5.44)	
**Anxiety/depression**	No problems	2 (10.00)	9 (3.54)	4 (2.01)	2 (1.96)	1 (3.13)	18 (2.97)	
	Slight problems	5 (25.00)	47 (18.50)	26 (13.07)	12 (11.76)	3 (9.38)	93 (15.32)	0.207
	Moderate problems	8 (40.00)	92 (36.22)	73 (36.68)	35 (34.31)	10 (31.25)	218 (35.91)	
	Severe problems	4 (20.00)	62 (24.41)	55 (27.64)	35 (34.31)	7 (21.88)	163 (26.85)	
	Unable/extreme problems	1 (5.00)	44 (17.32)	41 (20.60)	18 (17.65)	11 (34.38)	115 (18.95)	

Probability value (p value) was derived using Fisher's exact chi-squared test. *na* Not available

A notable percentage of survivors (45.80%) encountered severe or extreme problems of anxiety/depression. Additionally, approximately 21% reported experiencing severe or extreme pain/discomfort, with 19% facing challenges in their daily activities. Across all EQ-5D-5L dimensions—specifically, mobility, self-care, usual activities, and pain/discomfort—a statistically significant association with cancer stage was established (p < 0.05), highlighting the effects of cancer progression on HRQoL outcomes.

### Distribution of HRQoL

Table 3 shows EQ-5D-5L scores, showing that cancer site and stage are associated with HRQoL scores. Notably, HRQoL mean scores were lower among survivors afflicted with throat [mean = 0.46, (SD = 0.33)], brain [0.40, (0.36)], lung [0.46, (0.35)], blood [0.48, (0.32)], and liver cancer [0.48, (0.32)] in contrast to those grappling with breast [0.65, (0.25)], pancreas [0.65, (0.39)], oral [0.61, (0.24)], and cervical cancer [0.59 (0.31)]. HRQoL scores decreased with advanced cancer stages and were significantly lower for certain types of cancer. Private health facility and healthy lifestyle factors were associated with higher HRQoL scores. HRQoL scores tend to increase with higher education levels, affluent income brackets, smaller family sizes, and urban residence.

**Table 3:** The comparison of EQ-5D-5L utility scores by tumor stages

Patients' Characteristics	Mean EQ-5D Health Utility Scores (Standard Deviation, SD)						
	**Overall Mean Score (SD)**	**95% CI**	**Stage-0**	**Stage-I**	**Stage-II**	**Stage-III**	**Stage-IV**
**Tumor site**							
Oral	0.61 (0.24)	(0.55 to 0.67)	0.68 (0.21)	0.60 (0.25)	0.63 (0.18)	0.74 (0.16)	0.47 (0.39)
Breast	0.65 (0.25)	(0.60 to 0.69)	na	0.68 (0.24)	0.61 (0.25)	0.67 (0.24)	0.61 (0.37)
Lung	0.46 (0.35)	(0.38 to 0.55)	0.24 (0.22)	0.50 (0.37)	0.44 (0.35)	0.48 (0.32)	0.34 (0.70)
Blood	0.48 (0.32)	(0.30 to 0.66)	0.63 (0.19)	0.23 (0.60)	0.39 (0.39)	0.63 (0.06)	na
Pancreas	0.65 (0.39)	(0.43 to 0.86)	0.86 (0.19)	0.70 (0.26)	0.81 (0.12)	0.78 (0.20)	0.20 (0.64)
Liver	0.48 (0.32)	(0.34 to 0.62)	0.41 (0.22)	0.48 (0.34)	0.40 (0.41)	0.64 (0.16)	0.59 (0.21)
Cervix	0.59 (0.31)	(0.54 to 0.65)	0.73 (0.24)	0.60 (0.30)	0.51 (0.34)	0.68 (0.25)	0.63 (0.31)
Brain	0.40 (0.36)	(0.19 to 0.60)	0.84 (0.23)	0.39 (0.38)	0.35 (0.35)	0.35 (0.47)	na
Throat	0.46 (0.33)	(0.30 to 0.63)	0.85 (0.01)	0.54 (0.33)	0.43 (0.33)	0.06 (0.15)	0.30 (0.31)
Other tumor sites	0.56 (0.32)	(0.52 to 0.61)	0.81 (0.15)	0.53 (0.34)	0.62 (0.27)	0.57 (0.32)	0.22 (0.36)
**Ongoing treatment facility**							
Public facility	0.55 (0.32)	(0.52 to 0.58)	0.70 (0.26)	0.56 (0.31)	0.55 (0.30)	0.51 (0.34)	0.27 (0.41)
Private facility	0.72 (0.18)	(0.68 to 0.76)	0.41 (0.21)	0.88 (0.04)	0.77 (0.14)	0.72 (0.14)	0.66 (0.27)
**Age, years**							
18 to 35	0.49 (0.36)	(0.41 to 0.57)	0.74 (0.21)	0.47 (0.37)	0.45 (0.38)	0.59 (0.29)	0.19 (0.44)
36 to 45	0.59 (0.30)	(0.54 to 0.64)	0.67 (0.19)	0.61 (0.32)	0.57 (0.28)	0.59 (0.29)	0.57 (0.45)
46 to 64	0.58 (0.29)	(0.54 to 0.61)	0.71 (0.29)	0.56 (0.29)	0.60 (0.27)	0.61 (0.31)	0.49 (0.34)
> 64	0.58 (0.31)	(0.53 to 0.64)	0.60 (0.42)	0.61 (0.30)	0.56 (0.30)	0.60 (0.25)	0.25 (0.52)
**Gender**							
Male	0.54 (0.32)	(0.50 to 0.57)	0.63 (0.31)	0.56 (0.32)	0.54 (0.30)	0.51 (0.30)	0.31 (0.42)
Female	0.60 (0.30)	(0.57 to 0.63)	0.77 (0.15)	0.58 (0.31)	0.58 (0.29)	0.67 (0.26)	0.54 (0.37)
**Marital status**							
Never married	0.48 (0.39)	(0.32 to 0.63)	0.78 (0.13)	0.33 (0.38)	0.54 (0.40)	0.79 (0.19)	0.19 (0.44)
Married	0.58 (0.30)	(0.55 to 0.60)	0.72 (0.24)	0.58 (0.31)	0.56 (0.29)	0.61 (0.28)	0.46 (0.39)
Widowed/divorced/seperated	0.54 (0.34)	(0.44 to 0.64)	0.43 (0.36)	0.57 (0.29)	0.55 (0.35)	0.45 (0.46)	0.49 (0.73)
**Education**							
No education	0.54 (0.30)	(0.51 to 0.58)	0.45 (0.40)	0.56 (0.30)	0.52 (0.28)	0.58 (0.32)	0.4 (0.39)
Primary	0.59 (0.30)	(0.55 to 0.64)	0.68 (0.27)	0.58 (0.31)	0.62 (0.29)	0.58 (0.30)	0.52 (0.31)
Secondary	0.58 (0.34)	(0.51 to 0.65)	0.83 (0.12)	0.55 (0.37)	0.59 (0.32)	0.67 (0.15)	0.42 (0.50)
Higher secondary	0.59 (0.33)	(0.50 to 0.69)	0.85 (0.01)	0.53 (0.32)	0.68 (0.29)	0.81 (0.10)	0.37 (0.49)
Tertiary	0.65 (0.30)	(0.54 to 0.76)	0.55 (0.12)	0.72 (0.30)	0.48 (0.41)	0.63 (0.22)	0.61 (0.28)
**BMI Group**							
Underweight: < 18.49	0.46 (0.34)	(0.39 to 0.52)	0.81 (0.15)	0.42 (0.36)	0.51 (0.27)	0.49 (0.32)	0.34 (0.29)
Healthy weight: 18.50 to 24.99	0.56 (0.31)	(0.53 to 0.59)	0.64 (0.29)	0.57 (0.30)	0.54 (0.30)	0.58 (0.29)	0.47 (0.38)
Overweight: 25.00 to 29.99	0.69 (0.21)	(0.65 to 0.74)	0.77 (0.13)	0.71 (0.17)	0.67 (0.23)	0.67 (0.26)	0.74 (0.19)
Obese: ≥ 30	0.63 (0.33)	(0.54 to 0.73)	na	0.63 (0.35)	0.57 (0.35)	0.83 (0.10)	0.48 (0.45)
**Residence**							
Rural	0.57 (0.31)	(0.54 to 0.59)	0.70 (0.26)	0.57 (0.31)	0.55 (0.30)	0.59 (0.30)	0.45 (0.42)
Urban	0.60 (0.31)	(0.53 to 0.67)	0.46 (0.22)	0.58 (0.35)	0.63 (0.29)	0.73 (0.17)	0.40 (0.36)
**Family size**							
< 4 members	0.60 (0.27)	(0.54 to 0.66)	0.73 (0.38)	0.63 (0.29)	0.63 (0.23)	0.64 (0.20)	0.30 (0.31)
4 to 5 members	0.58 (0.31)	(0.55 to 0.62)	0.71 (0.23)	0.59 (0.31)	0.57 (0.29)	0.60 (0.29)	0.46 (0.42)
≥ 6 members	0.54 (0.32)	(0.50 to 0.59)	0.62 (0.33)	0.53 (0.32)	0.54 (0.32)	0.59 (0.33)	0.59 (0.50)
**Occupation**							
Unemployed	0.46 (0.34)	(0.41 to 0.52)	0.37 (0.20)	0.52 (0.31)	0.46 (0.35)	0.31 (0.38)	0.21 (0.37)
Employed	0.61 (0.30)	(0.52 to 0.70)	0.72 (0.22)	0.64 (0.27)	0.49 (0.40)	0.69 (0.24)	0.61 (0.20)
Business	0.69 (0.19)	(0.64 to 0.75)	0.71 (0.42)	0.71 (0.22)	0.67 (0.20)	0.69 (0.12)	0.76 (0.22)
Housewife	0.62 (0.28)	(0.58 to 0.65)	0.81 (0.10)	0.57 (0.32)	0.62 (0.25)	0.72 (0.19)	0.57 (0.36)
Informal worker	0.55 (0.27)	(0.47 to 0.63)	0.65 (0.25)	0.60 (0.27)	0.56 (0.19)	0.36 (0.33)	na
Students	0.50 (0.41)	(0.28 to 0.72)	0.73 (0.15)	0.39 (0.48)	0.56 (0.29)	0.79 (0.19)	0.29 (0.21)
Other occupations	0.54 (0.34)	(0.46 to 0.61)	0.67 (0.31)	0.57 (0.35)	0.55 (0.32)	0.52 (0.28)	0.24 (0.43)
**Income quintile**							
Q1 (20% lowest)	0.52 (0.33)	(0.47 to 0.56)	0.63 (0.30)	0.53 (0.34)	0.52 (0.30)	0.50 (0.34)	0.40 (0.42)
Q2	0.48 (0.32)	(0.39 to 0.56)	0.52 (0.35)	0.52 (0.29)	0.41 (0.34)	0.37 (0.34)	0.92 (0.32)
Q3	0.60 (0.30)	(0.54 to 0.65)	0.77 (0.20)	0.63 (0.27)	0.57 (0.29)	0.62 (0.31)	0.33 (0.49)
Q4	0.59 (0.31)	(0.53 to 0.64)	0.85 (0.14)	0.56 (0.32)	0.60 (0.28)	0.71 (0.23)	0.11 (0.39)
Q5 (20% highest)	0.67 (0.23)	(0.62 to 0.71)	0.76 (0.12)	0.63 (0.27)	0.71 (0.25)	0.66 (0.19)	0.66 (0.16)
**Received COVID-19 vaccine**							
No	0.53 (0.32)	(0.49 to 0.58)	0.41 (0.36)	0.53 (0.37)	0.50 (0.31)	0.62 (0.25)	0.36 (0.39)
Yes	0.58 (0.30)	(0.55 to 0.61)	0.70 (0.26)	0.58 (0.30)	0.59 (0.29)	0.58 (0.32)	0.49 (0.41)
**Smoking history**							
No	0.58 (0.31)	(0.55 to 0.61)	0.81 (0.15)	0.56 (0.32)	0.57 (0.30)	0.64 (0.27)	0.46 (0.41)
Yes	0.55 (0.30)	(0.50 to 0.60)	0.46 (0.29)	0.59 (0.28)	0.55 (0.29)	0.48 (0.34)	0.37 (0.39)
**Smokeless tobacco use**							
No	0.58 (0.31)	(0.54 to 0.61)	0.75 (0.23)	0.59 (0.32)	0.55 (0.31)	0.62 (0.27)	0.40 (0.39)
Yes	0.56 (0.31)	(0.52 to 0.60)	0.60 (0.29)	0.55 (0.31)	0.58 (0.29)	0.56 (0.32)	0.49 (0.42)
**Physical activity** (walk > 150 m/week)							
No	0.41 (0.36)	(0.36 to 0.47)	0.26 (0.31)	0.29 (0.35)	0.47 (0.34)	0.55 (0.32)	0.31 (0.44)
Yes	0.64 (0.26)	(0.61 to 0.66)	0.74 (0.21)	0.66 (0.24)	0.59 (0.28)	0.66 (0.24)	0.57 (0.33)

***Abbreviations****: BMI* Body Mass Index, *SD* Standard Deviation, *CI* Confidence Interval, *na* Not available

We found that HRQoL scores decrease with the progression of cancer stages. For instance, survivors confronting throat cancer displayed notably higher HRQoL scores at an early stage compared to an advanced stage (stage 0 = 0.85 vs. stage IV = 0.30). This trend persisted among survivors with brain cancer (stage 0 = 0.84 vs. stage III = 0.35) and breast cancer (stage I = 0.68 vs. stage IV = 0.61). Comparable patterns emerged for pancreatic (stage 0 = 0.86 vs. stage IV = 0.20), oral (stage 0 = 0.68 vs. stage IV = 0.47), and cervical cancer survivors (stage 0 = 0.73 vs. stage IV = 0.63). Important differences surfaced concerning HRQoL scores attributed to treatment facilities, where mean scores were markedly higher among patients treated in a private health facility (0.72 vs. public hospital = 0.55).

### Factors influencing health utility scores

The outcomes of the tobit regression model, presented in Table 4, revealed the important predictors influencing health utility scores. We found a statistically significant decrement in HRQoL scores among survivors with advanced-stage cancer (stage IV) (β = -0.49, 95% CI: -0.75 to -0.22) compared to patients with early-stage cancer (stage 0). Additionally, physically inactive cancer survivors had significantly lower utility scores (β = -0.41, 95% CI: -0.51 to -0.30) compared to their physically active counterparts. Furthermore, patients receiving treatment in private healthcare showed significantly higher utility scores (β = 0.35, 95% CI: 0.23 to 0.48) relative to those treated in public hospitals. No significant association was found between HRQoL scores and cancer site, gender, education, marital status, residence, or family composition.

**Table 4:** Association between patients’ characteristics and health-related quality of life

Patients’ characteristics	Model-1 (Unadjusted)		Model-2 (Adjusted)	
	**β coeff (95% CI)**	***p*-value**	**β coeff (95% CI)**	***p*-value**
**Tumor site** (ref = other tumor sites)				
Oral	0.08 (-0.06 to 0.22)	0.279	0.08 (-0.05 to 0.21)	0.229
Breast	0.14 (0.02 to 0.26)	0.025	0.09 (-0.04 to 0.21)	0.160
Lung	-0.20 (-0.37 to -0.02)	0.029	-0.13 (-0.28 to 0.02)	0.090
Blood	-0.16 (-0.52 to 0.21)	0.399	-0.15 (-0.46 to 0.16)	0.353
Pancreas	0.14 (-0.13 to 0.41)	0.301	0.16 (-0.07 to 0.40)	0.166
Liver	-0.16 (-0.44 to 0.12)	0.262	-0.16 (-0.39 to 0.08)	0.197
Cervix	0.05 (-0.07 to 0.17)	0.429	0.08 (-0.04 to 0.19)	0.197
Brain	-0.36 (-0.80 to 0.09)	0.114	-0.23 (-0.59 to 0.14)	0.221
Throat	-0.20 (-0.53 to 0.13)	0.242	-0.03 (-0.31 to 0.25)	0.824
**Tumor stage** (ref = Stage-0)				
Stage-I	-0.19 (-0.40 to 0.02)	0.073	-0.11 (-0.30 to 0.08)	0.252
Stage-II	-0.20 (-0.41 to 0.01)	0.061	-0.17 (-0.37 to 0.02)	0.086
Stage-III	-0.13 (-0.35 to 0.09)	0.231	-0.12 (-0.33 to 0.09)	0.260
Stage-IV	-0.45 (-0.76 to -0.13)	0.005	-0.49 (-0.75 to -0.22)	**<0.001**
**Ongoing treatment facility: private facility** (ref = public facility)	0.27 (0.17 to 0.38)	<0.001	0.35 (0.23 to 0.48)	**<0.001**
**Received COVID-19 vaccine** (ref = no)	0.09 (-0.01 to 0.19)	0.081	0.05 (-0.04 to 0.14)	0.252
**Smoking status** (ref = no)	-0.05 (-0.15 to 0.05)	0.348	0.11 (-0.01 to 0.23)	0.076
**Smokeless tobacco use** (ref = no)	-0.03 (-0.12 to 0.05)	0.454	-0.01 (-0.09 to 0.07)	0.775
**Physical activity: > 150 min per week** (ref = yes)	-0.43 (-0.54 to -0.32)	<0.001	-0.41 (-0.51 to -0.30)	**<0.001**
**BMI** (ref = healthy weight: 18.50 to 24.99)				
Underweight: < 18.49	-0.21 (-0.35 to -0.07)	0.004	-0.06 (-0.19 to 0.06)	0.323
Overweight: 25.00 to 29.99	0.21 (0.10 to 0.31)	<0.001	0.13 (0.03 to 0.22)	**0.012**
Obese: ≥ 30	0.12 (-0.03 to 0.27)	0.121	0.07 (-0.07 to 0.21)	0.344
**Urban community** (ref = rural)	0.06 (-0.07 to 0.19)	0.346	-0.03 (-0.15 to 0.08)	0.563
**Family size** (ref = < 4 members)				
4 to 5 members	-0.04 (-0.17 to 0.09)	0.581	-0.04 (-0.16 to 0.08)	0.520
≥ 6 members	-0.10 (-0.24 to 0.04)	0.146	-0.06 (-0.19 to 0.06)	0.317
**Occupation** (ref = unemployed)				
Employed	0.28 (0.09 to 0.47)	0.004	0.07 (-0.11 to 0.25)	0.423
Businessmen	0.40 (0.23 to 0.57)	<0.001	0.18 (0.01 to 0.35)	**0.040**
Housewife	0.29 (0.17 to 0.41)	<0.001	0.10 (-0.04 to 0.23)	0.168
Informal worker	0.17 (-0.03 to 0.37)	0.092	0.11 (-0.07 to 0.29)	0.221
Students	0.08 (-0.26 to 0.42)	0.644	0.05 (-0.30 to 0.39)	0.784
Other occupations	0.14 (-0.02 to 0.30)	0.076	0.08 (-0.07 to 0.22)	0.297
**Income quintile** (ref = Q5: 20% highest)				
Q1 (20% lowest)	-0.26 (-0.37 to -0.14)	<0.001	-0.14 (-0.25 to -0.03)	**0.016**
Q2	-0.33 (-0.53 to -0.14)	0.001	-0.31 (-0.49 to -0.14)	**<0.001**
Q3	-0.11 (-0.24 to 0.02)	0.089	-0.03 (-0.15 to 0.09)	0.656
Q4	-0.13 (-0.25 to -0.01)	0.045	-0.07 (-0.18 to 0.04)	0.203
**Age, years** (ref = 18 to 35 years)				
36 to 45	0.19 (0.03 to 0.35)	0.021	0.19 (0.05 to 0.34)	**0.010**
46 to 64	0.17 (0.02 to 0.32)	0.027	0.20 (0.06 to 0.34)	**0.006**
> 64	0.17 (0.01 to 0.34)	0.038	0.25 (0.09 to 0.41)	**0.002**
**Female patients** (ref = male)	0.11 (0.02 to 0.20)	0.013	0.09 (-0.07 to 0.25)	0.258
**Marital status** (ref = never married)				
Married	0.19 (-0.07 to 0.45)	0.157	-0.23 (-0.50 to 0.05)	0.103
Widowed/divorced/seperated	0.12 (-0.18 to 0.43)	0.434	-0.25 (-0.55 to 0.05)	0.106
**Education** (ref = tertiary)				
No education	-0.18 (-0.37 to 0.01)	0.059	-0.11 (-0.30 to 0.08)	0.263
Primary	-0.09 (-0.28 to 0.11)	0.383	0.01 (-0.17 to 0.20)	0.914
Secondary	-0.10 (-0.31 to 0.10)	0.319	-0.06 (-0.25 to 0.13)	0.520
Higher secondary	-0.09 (-0.32 to 0.14)	0.451	-0.02 (-0.23 to 0.18)	0.814

***Abbreviations***: *β coeff* β coefficient, *CI* Confidence interval, *p-value* Probability value, *ref* Reference group

## Discussion

The current study examined HRQoL within the cohort of cancer survivors in Bangladesh, offering insights into the dynamic interplay between cancer site, stage, and HRQoL trajectories during systemic and radiation therapy. Findings revealed that HRQoL scores differ for cancer survivors based on cancer sites and stages. For instance, the most diminished scores were consistently identified among patients confronted by advanced-stage (stage IV) cancer. Notably, our analysis further underscores the distinct HRQoL profiles associated with varying cancer sites, with throat, brain, lung, blood, and liver cancer survivors encountering markedly lower HRQoL compared to their counterparts diagnosed with breast, pancreatic, oral, or cervical cancer. By substantiating these associations, our study contributes to a more complete understanding of the intricate relationship between cancer characteristics and patients' quality of life outcomes.

Our study has revealed that cancer survivors with advanced-stage (stage IV) had significantly lower HRQoL scores by 0.49 units compared to those who were early-stage (stage-0) survivors. The findings are consistent with prior studies on the relationship between cancer stage and HRQoL, reporting that advanced-stage cancers significantly correlated with worse HRQoL [30, 31]. They also align with the results of a recent meta-analysis that has revealed that patients with stage IV cancer had a 4.92 times higher chance of developing poor HRQoL than those with others [32]. A recent study, however, found that HRQoL was not associated with cancer stage [33]. There are several possible explanations for our findings of lower HRQoL scores among stage-IV cancer patients compared to those with stage-0 cancer. First, this inconsistency according to stage is probably due to the different clinical characteristics of cancers; specifically, potential adverse side effects of the disease are the main domains of HRQoL in various stages of the disease, from non-invasive cancer to a tendency to develop a more advanced-stage (stage IV) cancer [11]. Further research is needed to understand the disproportionate decrease in HRQoL among cancer survivors. The authors urge more awareness of HRQoL cancer survivors and the need to address poor HRQoL in the clinical settings. Second, it is possible that much of the variation in HRQoL occurs during systemic therapy and radiotherapy. This is because HRQoL often falls during the treatment phase due to more fatigue, nausea and vomiting, loss of appetite, and systemic side effects, whereas advanced-stage patients suffer significant functional limitations related to the condition [9, 33, 34]. In other words, HRQoL for patients with advanced-stage cancer is likely affected by the burden of the disease itself and the treatment regimens applied to the disease [7, 33, 35]. Normally, cancer patients with stage 0-II undergo surgery and some receive additional therapy, but patients with stage III & IV cancer most often receive radiation therapy and/or chemotherapy in addition to surgery. Patients undergoing treatment experience a decline in their perceived HRQoL during treatment. It could also indicate a decrease in HRQoL may reflect changes in treatment patterns for late-stage cancers. Therefore, the authors suggest not only a role for multidisciplinary rehabilitation for patients after treatment but also rehabilitation before treatment. Last, it could be due to the fact that the more advanced stage is used as a proxy marker for a poor outcome since the more advanced stage is related to lower HRQoL [9, 10]. In other words, some functional disorders that occur as a result of particular types of treatment, like chemotherapy and immunotherapy, are more likely to result in worse HRQoL [36]. It is pertinent to mention that we assessed the HRQoL of those undergoing systemic therapy and radiotherapy, so there is a high risk of having adverse effects on HRQoL. This is owing to the fact that if the disease is not appropriately treated and controlled, it will worsen over time [31]. Therefore, it is crucial to diagnose cancer at earlier stages for a better treatment outcome and HRQoL.

Specifically, patients with stage IV cancer more commonly reported severe or extreme problems across all EQ-5D domains compared to the first three stages of cancer. Similar results were found in a recent study conducted in Korea has revealed that most cancer patients had severe difficulties in all EQ-5D dimensions [37]. Further, the most frequently reported dimensional problems among cancer survivors were anxiety/depression and pain/discomfort. Conversely, a previous study reported a highly prevalent dimensional problem among cancer survivors were pain/discomfort and mobility [37]. Gao et al. [25] observed that 54.6% of patients reported pain/discomfort, and 41.2% reported anxiety or depression. Conversely, our findings reported 21% of patients suffered from pain/discomfort and 49.1% had anxiety/depression. A possible explanation could be that advanced-stage cancer patients experience a range of symptoms for which standard medical treatments may not provide sufficient relief rather than that systemic therapy and radiotherapy could be contributing to lower HRQoL. In clinical settings, healthcare providers should endeavor to manage these problems to mitigate patients’ symptoms, pain/discomfort, and anxiety/depression by implementing tailored interventions to meet patients’ needs during treatment and when providing healthcare services such as palliative care, particularly for patients at advanced cancer stages.

We found that the HRQoL of cancer survivors differed depending on the cancer site; for example, patients with throat, brain, lung, blood and liver cancer had a lower HRQoL compared to those with breast, pancreatic, oral, and cervical cancer. In a previous study, a similar trend was observed between cancer sites; for example, breast and genital cancer survivors expressed slightly higher HRQoL mean scores than those with cancer in the digestive system [20]. In addition, our study did not find any statistically significant correlation between cancer sites and HRQoL. Contrary to a recent study in Korea, which has shown a statistically significant association between cancer sites and HRQoL [37]. Even though we did not find any significant association between cancer site and HRQoL, it has clinical importance. Therefore, we suggest conducting further studies to identify factors associated with HRQoL differences between cancer sites.

According to our study data, patients who received treatment in a private hospital were more likely to attain better HRQoL compared to those in a public hospital. The findings are supported by another study that revealed that cancer patients treated in private hospitals had a comparatively better HRQoL due to better facilities, advanced treatment modalities, and technologies [38]. There is a significant difference between the cost of care in government-facilitated centers and private centers, which might affect the quality of care as well as the quality of life. In Bangladesh, the public sector provides not-for-profit services that include preventive, curative, promotional, and rehabilitative services. In contrast, the private sector providers are mostly for-profit curative services that operate in urban areas, mainly in the capital city of Dhaka and other major cities. Due to the high cost of private hospitals, most people can’t afford to go there for treatment [39]. The private sector hospitals give the highest priority to serving their patients and try to employ more providers than the public sector. Alarmingly, many doctors employed in the public sector of Bangladesh are sometimes engaged in the private sector as well, which reduces the service capacity of public hospitals [40]. Therefore, the respective authorities should revisit cancer centers for quality care and increase the capacity to afford more cancer patients in public healthcare facilities. The government can ban joint public/private practices because they interfere with public sector efficiency, as well as increase allowances and incentives for public sector employees to ensure quality care. The authors propose the urgent need to formulate a National Cancer Policy encompassing early detection, affordable care, and comprehensive supportive care through well-defined care pathways at public health facilities.

This study has several important clinical, research and policy implications. The current study highlights the declining HRQoL of cancer patients. With treatment and care now becoming more patient-centered, it has become more pertinent to understand the impact of cancer treatments in the advanced stages on the HRQoL of patients. The effect of treatment for certain cancers may be extreme and may involve a great deal of HRQoL. Therefore, the authors stress the importance of conducting research to observe changes in symptoms and HRQoL among cancer patients at different stages of the disease. To better understand how HRQoL varies among cancer stages, future research could be initiated to examine patient HRQoL before, during, and after treatment.

### Strengths and limitations

Our study addresses an unexplored avenue within the field of cancer research in Bangladesh. To our knowledge, no prior investigations have undertaken an inclusive examination of HRQoL encompassing all cancer sites and stages during treatment. The study offers a comprehensive perspective by including patients from diverse geographical regions from the country's most prominent public and private cancer hospitals.

The present study, despite its contributions, has some limitations. Firstly, its cross-sectional design inherently poses the risk of selection bias, underscoring the need for a cautious interpretation of the results. Secondly, while the assessment of HRQoL during treatment provides valuable insights, the comprehensive evaluation could be enriched by encompassing pre-treatment and post-treatment stages within the same cohort. Moreover, the absence of EQ-VAS scores as a measure of HRQoL limits the comprehensive evaluation of this multifaceted construct. Further, reliance on self-reported cancer diagnosis, site, and treatment history may entail potential reporting biases. Lastly, the study's age criterion (18 years and above) excludes the exploration of HRQoL among cancer patients below 18 years old, signifying a scope for further research to encompass this demographic segment.

## Conclusion

The findings highlight the significant influence of cancer stage and site on survivors' overall HRQoL, with those with advanced-stage disease reporting lower HRQoL scores compared to early-stage survivors. Specifically, survivors of brain, throat, lung, blood, and liver cancer appear to face greater challenges in maintaining HRQoL. Moreover, findings revealed that physically inactive patients with advanced-stage cancer experienced notably lower HRQoL utility scores. This highlights the potential benefits of incorporating physical activity into cancer care strategies, particularly for those facing advanced disease. Survivors who received treatment in a private hospital reported higher HRQoL utility scores compared to their counterparts treated in a public hospital. This observation raises questions about potential disparities in care quality in health facilities and suggests avenues for targeted improvements.

The study emphasises the importance of routine HRQoL assessments for cancer patients, offering a valuable tool for identifying individuals at risk of poor HRQoL outcomes. This proactive approach enables clinicians to tailor interventions, potentially enhancing patients' overall well-being. To facilitate future research on HRQoL among cancer survivors, more cancer centers and an equal number of data from public and private health facilities must be included for better findings.

### Supplementary Information

**Additional file 1. **

## Data Availability

The data used and/or analysed during the current study are available upon reasonable request from the corresponding author.

## Abbreviations

HRQoL: Health-related quality of life
LIMCs: Low- and middle-income countries
SDG: Sustainable Development Goal
SD: Standard deviation
EQ-5D-5L: EuroQol-5D-5L
