# Supplementary figures and images for: Assessing health-related quality of life among cancer survivors during systemic and radiation therapy in Bangladesh: a cancer-specific exploration
Source: BMC Cancer. 2023 Dec 7;23:1208. doi: 10.1186/s12885-023-11670-z (PMC10704718; doi:10.1186/s12885-023-11670-z)

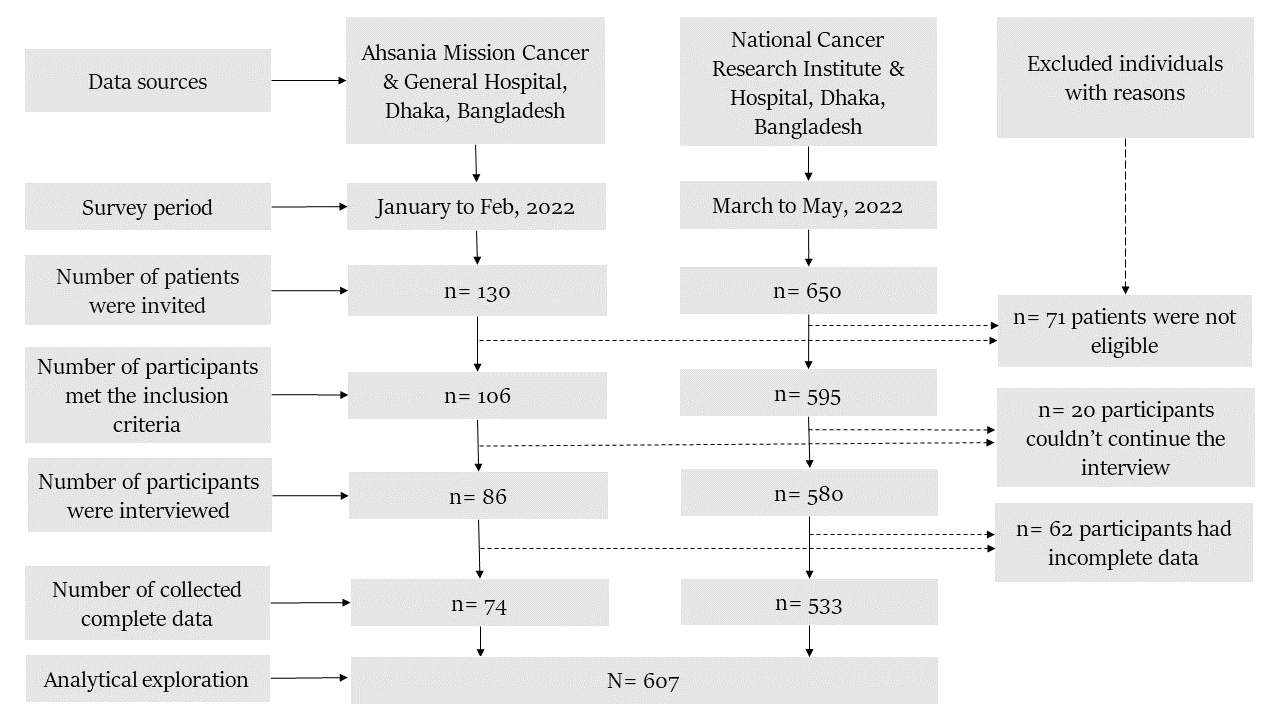

Supplement: Supplementary file 1 — Additional file 1. [file 12885_2023_11670_MOESM1_ESM.png]
